# Supplementary material for: Conformations of Bcs1L undergoing ATP hydrolysis suggest a concerted translocation mechanism for folded iron-sulfur protein substrate
Source: Nat Commun. 2024 May 31;15:4655. doi: 10.1038/s41467-024-49029-y (PMC11143374; doi:10.1038/s41467-024-49029-y)
Supplement: Supplementary file 1 — Supplementary Information [file 41467_2024_49029_MOESM1_ESM.pdf]

Supplementary Information

Supplementary Table 1

Supplementary Table 1. Measurement of substrate-stimulated ATPase activity for mBcs1L.

**Bcs1** (μg)	**ISP-ED** (μg)	**ISP-ED:Bcs1-7mer** molar ratio[a]	**ATPase Activity (nmole Pi/nmol monomer/min)** assay 1	assay 2	assay 3	Mean±σ	**% wild type**
4.0	0	0	7.843	7.722	7.858	7.81±0.07	100
4.0	0.28	1.7	10.044	10.071	10.095	10.07±0.02	129
4.0	0.56	3.4	10.881	10.246	12.542	11.22±1.19	144
4.0	0.84	5.1	10.354	10.570	10.514	10.48±0.11	134
4.0	1.12	6.8	9.720	10.408	10.137	10.09±0.35	129
4.0	1.40	8.5	9.477	9.598	9.606	9.56±0.07	122

a. ISP-ED has a molecular weight of 14.4 kDa and Bcs1 7mer has a molecular weight of 334 kDa.

Supplementary Table 2

Supplementary Table 2. Structural superpositions (RMSD in Å) between pairs of Bcs1 structures in indicated nucleotide states.[a]

	ATPγS (C7) (PDB:6UKS)	ATP-1 (C7) (PDB:8T5U)	ATP-1 (C1) (PDB:8TI0)	ATP-2 (C7) (PDB:8TP1)	ATP-2 (C1) (PDB:8TPL)	Apo (C7) (PDB:6UKP)	ADP (C7) (PDB:8T14)	ADP (C1) (PDB:8T7U)	Apo (C1) (PDB:8TBY)
**ATPγS (C7) (PDB:6UKS)**	-	0.644 (1,743)[b]	0.891 (1,806)	0.911 (2,338)	0.882 (2,321)	NA	NA	NA	NA
**ATP-1 (C7) (PDB:8T5U)**	0.421 (429)	-	0.285 (2,661)	1.306 (2,016)	1.268 (1,998)	NA	NA	NA	NA
**ATP-1 (C1) (PDB:8TI0)**	0.421 (429)	0.0 (357)	-	1.535 (2,058)	1.513 (2,054)	NA	NA	NA	NA
**ATP-2 (C7) (PDB:8TP1)**	0.505 (291)	0.705 (266)	0.705 (266)	-	0.149 (2,827)	NA	NA	NA	NA
**ATP-2 (C1) (PDB:8TPL)**	0.510 (296)	0.694 (266)	0.694 (266)	0.144 (407)	-	NA	NA	NA	NA
**Apo (C7) (PDB:6UKP)**	NA	NA	NA	NA	NA	-	1.058 (2,278)	1.072 (2,302)	1.222 (2,259)
**ADP (C7) (PDB:8T14)**	NA	NA	NA	NA	NA	0.834 (317)	-	0.248 (2,479)	1.515 (2,520)
**ADP (C1) (PDB:8T7U)**	NA	NA	NA	NA	NA	0.820 (325)	0.212 (342)	-	1.511 (2,523)
**Apo (C1) (PDB:8TBY)**	NA	NA	NA	NA	NA	0.899 (355)	0.899 (355)	0.884 (362)	-

a. numbers in top triangle are superposition of heptamers; numbers in bottom triangle are superposition of individual subunits.
b. numbers in parentheses are number of CA atoms superposed, with a cutoff distance of 2 Å.

Supplementary Table 3

Supplementary Table 3. BSA analysis of subunit association of mBcs1 under various nucleotide states[a]

	BSA (Å²)							
Subunit pair	Apo		ADP		ATP-1		ATP-2	
	*C*1[b]	*C*7 (6UKP)[c]	*C*1	*C*7	*C*1	*C*7	*C*1	*C*7
Range	25-420[d]	25-420[d]	1-420[d]	1-420[d]	47-417[d]	47-417[d]	1-419[d]	1-419[d]
AB	4,626	4,667	5,705<br>(5,638[e]; 4,567[f])	5,553<br>(5,463[e]; 4,626[f])	4,847[e]<br>(4,490[f])	4,791[e]<br>(4,600[f])	6,478<br>(6,319[e]; 5,585)	6,429<br>(6,290[e]; 5,567[f])
BC	4,603	4,667	5,728<br>(5,666; 4535)	5,626<br>(5,538; 4,658)	5,104<br>(4,663)	4,714<br>(4,524)	6,466<br>(6,306; 5,569)	6,413<br>(6,258; 5,549)
CD	4,594	4,747	5,635<br>(5,579; 4,505)	5,589<br>(5,499; 4,655)	5,063<br>(4,581)	4,735<br>(4,544)	6,452<br>(6,228; 5,596)	6,403<br>(6,247; 5,545)
DE	4,520	4,698	5,709<br>(5,652; 4,562)	5,587<br>(5,500; 4,638)	5,194<br>(4,737)	4,694<br>(4,501)	6,461<br>(6,300; 5,579)	6,402<br>(6,248; 5,545)
EF	4,654	4,658	5,718<br>(5,653; 4,544)	5,589<br>(5,501; 4,637)	4,879<br>(4,518)	4,725<br>(4,532)	6,525<br>(6,365; 5,629)	6,410<br>(6,255; 5,547)
FG	4,751	4,699	5,683<br>(5,624; 4,537)	5,590<br>(5,503; 4,635)	5,023<br>(4,542)	4,735<br>(4,535)	6,463<br>(6,303; 5,563)	6,425<br>(6,270; 5,559)
GA	4,317	4,650	5,696<br>(5,633; 4,522)	5,654<br>(5,564; 4,677)	5,128<br>(4,691)	4,849<br>(4,456)	6,484<br>(6,321; 5,584)	6,429<br>(6,276; 5,560)
<BSA>	4,581	4,684	5,696<br>(5,635; 4,539)	5,598<br>(5,510; 4,647)	5,034<br>(4,603)	4,749<br>(4,528)	6,476<br>(6,314; 5,586)	6,416<br>(6,263; 5,553)
RMSD	135	34	31<br>(28; 22)	32<br>(32; 17)	129<br>(95)	52<br>(44)	24<br>(25; 22)	12<br>(15; 9)

a. a probe of radius of 1.4 Å was used and BSA was calculated in CCP4.
b. *C*1 and *C*7 indicate structures have imposed C1 and C7 symmetry, respectively.
c. BSA numbers were calculated using previously deposited structure.
d. residues 1-47 is N-terminus+TMH; residues 48-420 is Bcs1 without TMH; residues 1-420 is the full-length Bcs1.
e. numbers in parentheses represent BSA with ADP or ATP removed.
f. numbers on the right column represent BSA with both ADP/ATP and the TMHs (residues 1-47) removed.

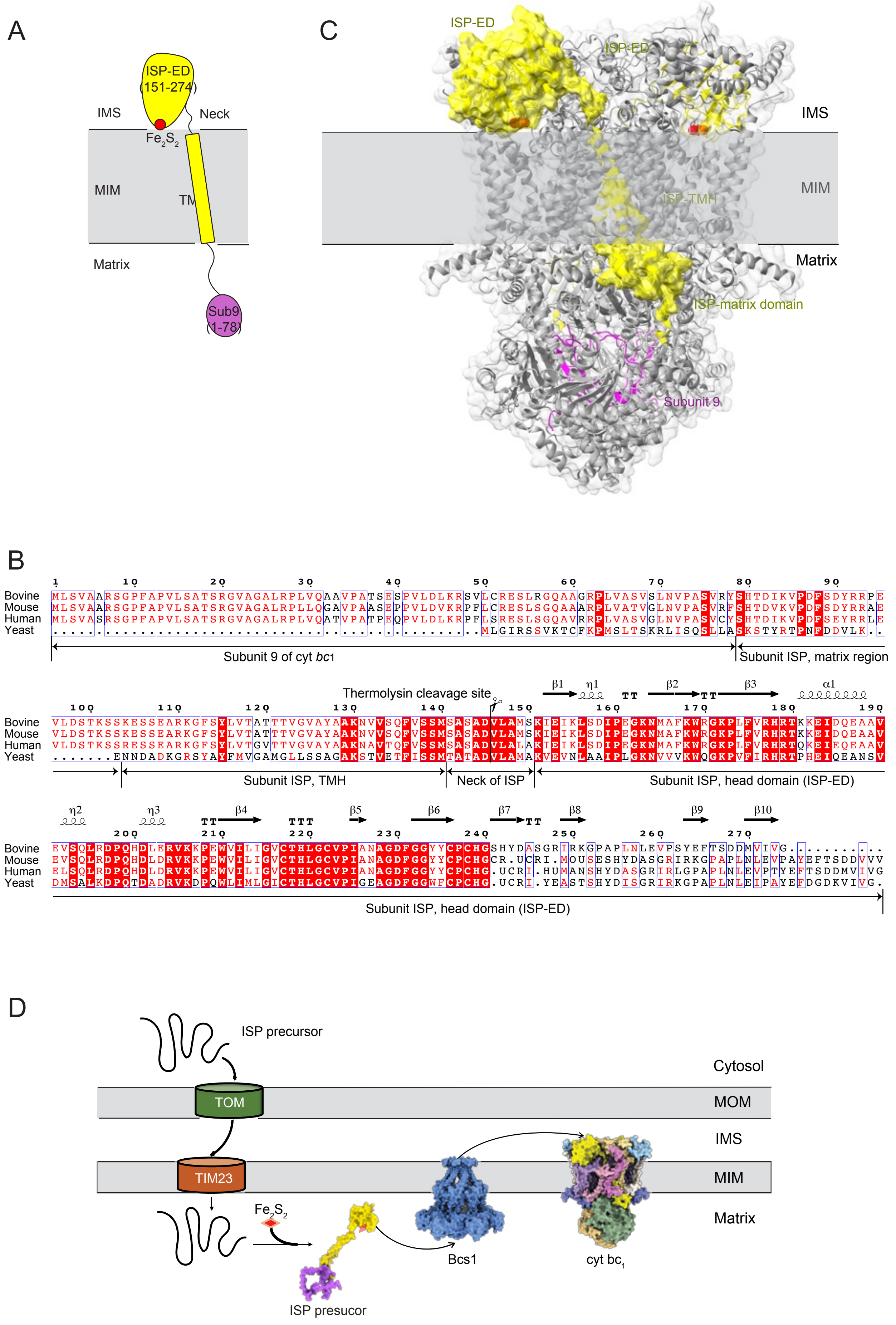
A
ISP-ED
(151-274)
IMS
Neck
Fe_2S_2
MIM
TM
Matrix
Sub9
(1-78)
C
ISP-ED
ISP-ED
IMS
MIM
ISP-TMH
Matrix
ISP-matrix domain
Subunit 9
B
1 10 20 30 40 50 60 70 80 90
Bovine MLSVAARSGPFAPVLSATSRGVAGALRPLVQAAVPATSESPVLDLKRSVLCRESLRGQAAGRPLVASVSLNVPASVRYSHTDIKVPDFSDYRRPE
Mouse MLSVAARSGPFAPVLSATSRGVAGALRPLLQGAVPAASEPPVLDVKRPFLCRESLSGQAAARPLVATVGLNVPASVRFSHTDVKVPDFSDYRRAE
Human MLSVASRSGPFAPVLSATSRGVAGALRPLVQATVPATPEQPVLDLKRPFLSRESLSGQAVRRPLVASVGLNVPASVCYSHTDIKVPDFSEYRRLE
Yeast ....MLGIRSSVKTCFKPMSLTSKRLISQSLLASKSTYRTPNFDDVLK..
Subunit 9 of cyt *bc*1
Subunit ISP, matrix region
Thermolysin cleavage site
β1 η1 TT β2 TT β3 α1
100 110 120 130 140 150 160 170 180 190
Bovine VLDSTKSSKESSEARKGFSYLVTATTTVGVAYAAKNVVSQFVSSMSASADVLAMSKIEIKLSDIPEGKNMAFKWRGKPLFVRHRTKKEIDQEAAV
Mouse VLDSTKSSKESSEARKGFSYLVTATTTVGVAYAAKNVVSQFVSSMSASADVLAMSKIEIKLSDIPEGKNMAFKWRGKPLFVRHRTKKEIDQEAAV
Human VLDSTKSSRESSEARKGFSYLVTGVTTVGVAYAAKNAVTQFVSSMSASADVLALAKIEIKLSDIPEGKNMAFKWRGKPLFVRHRTQKEIEQEAAV
Yeast ........ENNDADKGRSYAYFMVGAMGLLSSAGAKSTVETFISSMTATADVLAMAKVEVNLAAIPLGKNVVVKWQGKPVFIRHRTPHEIQEANSV
Subunit ISP, TMH
Neck of ISP
Subunit ISP, head domain (ISP-ED)
η2 η3 TT β4 TTT β5 β6 β7 TT β8 β9 β10
200 210 220 230 240 250 260 270
Bovine EVSQLRDPQHDLERVKKPEWVILIGVCTHLGCVPIANAGDFGGYYCPCHGSHYDASGRIRKGPAPLNLEVPSYEFTSDDMVIVG..........
Mouse EVSQLRDPQHDLDRVKKPEWVILIGVCTHLGCVPIANAGDFGGYYCPCHGCR.UCRI.MOUSESHYDASGRIRKGPAPLNLEVPAYEFTSDDVVV
Human ELSQLRDPQHDLDRVKKPEWVILIGVCTHLGCVPIANAGDFGGYYCPCHG.UCRI.HUMANSHYDASGRIRLGPAPLNLEVPTYEFTSDDMVIVG
Yeast DMSALKDPQTDADRVKDPQWLIMLGICTHLGCVPIGEAGDFGGWFCPCHG.UCRI.YEASTSHYDISGRIRKGPAPLNLEIPAYEFDGDKVIVG.
Subunit ISP, head domain (ISP-ED)
D
ISP precursor
Cytosol
TOM
MOM
IMS
TIM23
MIM
Fe_2S_2
Matrix
Bcs1
cyt bc_1
ISP presucor

Supplementary Figure 1. Sequence alignment of ISP precursors. (A) Cartoon representation of the ISP precursor protein as being presented to the core cyt bc_1 assembly. (B) Sequences of ISP precursors from different organisms including human, cow, mouse, and yeast were aligned. Unlike metazoan, Rip1 of yeast does not have a large N-terminal precursor sequence for subunit 9. Secondary structure elements for ISP-ED are indicated. (C) A cartoon representation of the structure of dimeric bovine cyt bc_1 (PDB:1NTM). All subunits are colored gray except for the two symmetry-related ISP subunits shown as surface models (yellow) and subunits 9 (magenta) in ribbon diagrams. After the ISP precursor is translocated by Bcs1, it is further processed into the ISP subunit and the subunit 9 of cyt bc_1 complex. (D) Proposed mechanism for ISP biogenesis and maturation.

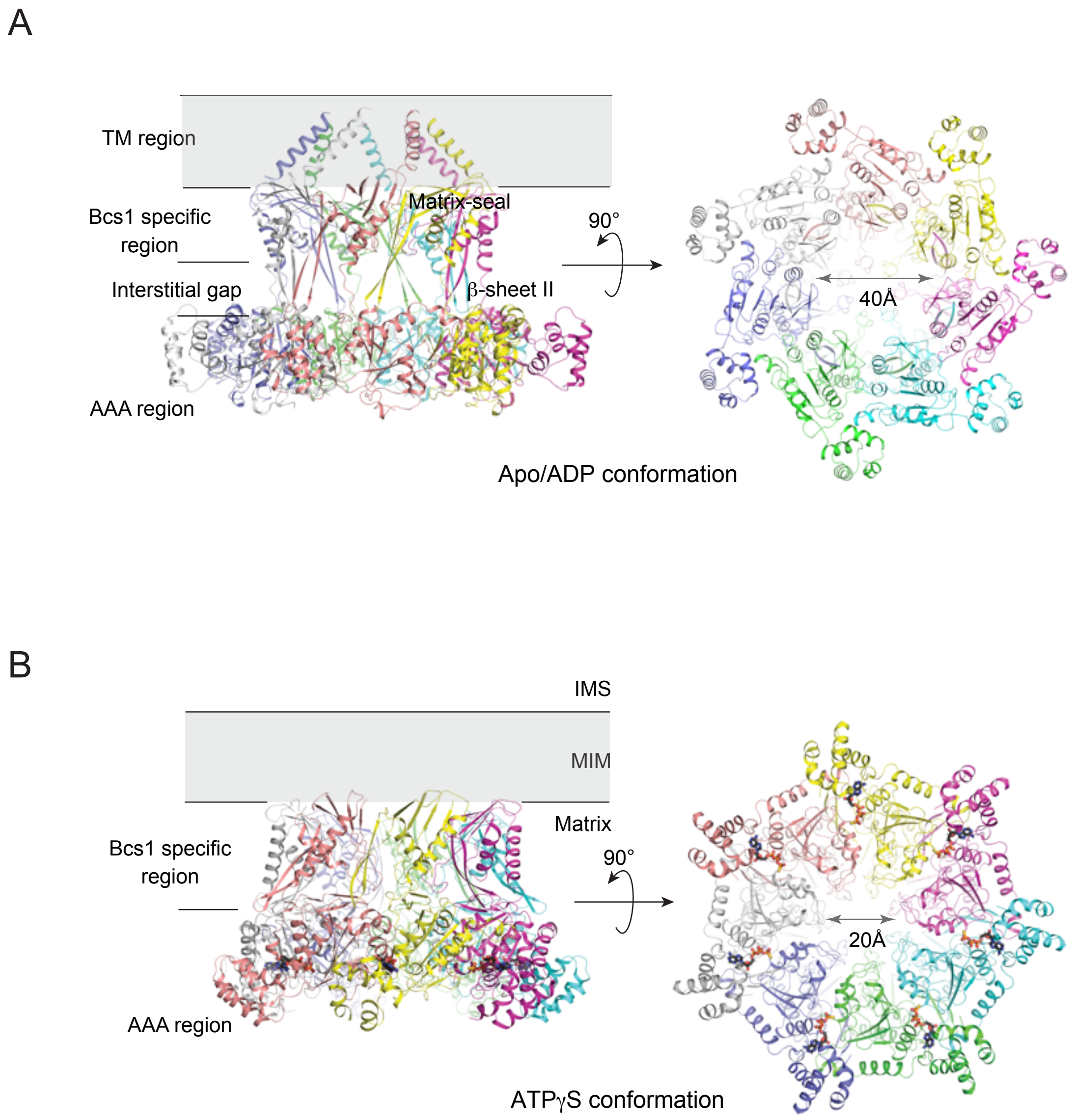
A
TM region
Bcs1 specific region
Interstitial gap
AAA region
Matrix-seal
β-sheet II
90°
40Å
Apo/ADP conformation
B
IMS
MIM
Matrix
Bcs1 specific region
AAA region
90°
20Å
ATPγS conformation

Supplementary Figure 2. Two known conformations for Bcs1. (A) Cartoon representation of the Apo/ADP conformation that was observed for mBcs1L and yeast Bcs1. Two orthogonal views were provided. The mitochondrial inner membrane is shown as a gray bar. The three regions of Bcs1 are indicated. The extended β-sheet II that props up the Bcs1-specific region to create the Interstitial gap were also indicated. This conformation also features a large entrance (40 Å in diameter) to the substrate binding cavity. (B) Cartoon rendition of the ATPγS conformation observed for mBcs1L. This conformation shows the disappearance of the Interstitial gap and a shrunken entrance to the substrate cavity (20 Å in diameter).

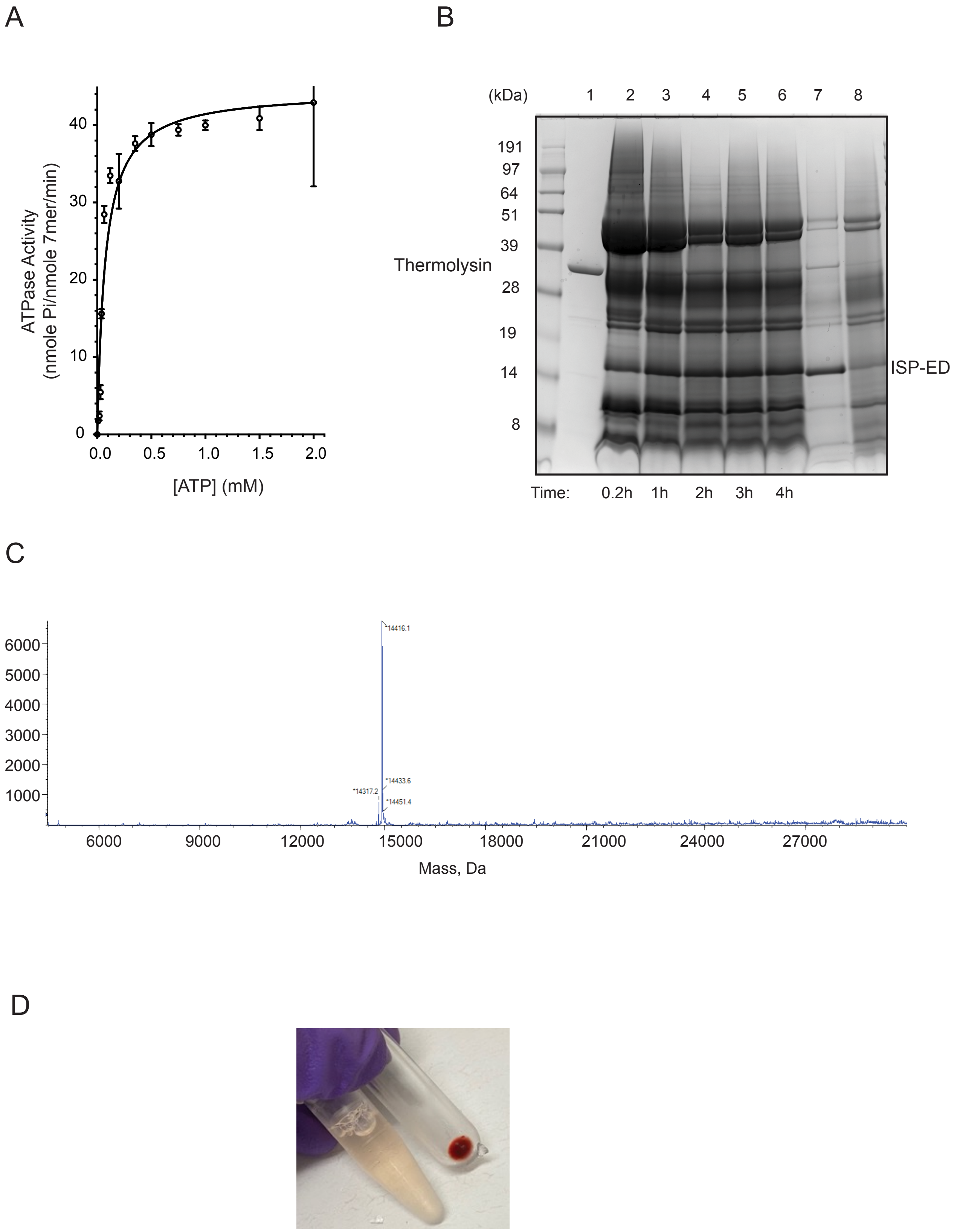
A
B
C
D
ATPase Activity
(nmole Pi/nmole 7mer/min)
[ATP] (mM)
0.0
0.5
1.0
1.5
2.0
0
10
20
30
40
(kDa)
1
2
3
4
5
6
7
8
191
97
64
51
39
28
19
14
8
Thermolysin
ISP-ED
Time:
0.2h
1h
2h
3h
4h
6000
5000
4000
3000
2000
1000
6000
9000
12000
15000
18000
21000
24000
27000
Mass, Da
14416.1
14433.6
14317.2
14451.4

Supplementary Figure 3. Characterization of mBcs1L ATPase activity and isolation of ISP-ED. (A) Basal ATPase activity in nmol Pi released/nmol of mBcs1L heptamer/min measured against concentrations of ATP. Measurements were carried out in biological triplets. The data points were fit with the Michaelis-Menten equation in Prism with a Vmax of 44.5 nmole Pi/nmol Bcs1 heptamer/min and Km of 0.073 mM. (B) Purification of ISP-ED from isolated bovine mitochondrial cyt bc_1. A 12% Bis-Tris SDS-PAGE gel was used for product analysis. Lane 1: thermolysin, lanes 2-6: treatment of cyt bc_1 complex by thermolysin for the indicated times, lane 7: centrifugation supernatant of the digested then dialyzed cyt bc_1, lane 8: precipitant of digested then dialyzed cyt bc_1. The molecular marker is shown on the left-most lane and labeled. (C) Mass-spectrometry (LC-MS) analysis with the deconvoluted ionization spectrum of isolated ISP-ED shows a major peak at a molecular weight of 14.4 *k*Da, which matches exactly the predicted MW of ISP-ED using the primary sequence after removing the 2 protons in cysteines. (D) Isolated bovine heart mitochondrial cyt bc_1 complex was treated with thermolysin and sampled in different time intervals from 0.2 to 4 hrs, followed by extensive dialysis to remove detergents in the solution. After dialysis, the cloudy protein solution was centrifuged. The supernatant contains ISP-ED that is straw-colored attributed to the bound Fe_2S_2 cluster and the insoluble cyt bc_1 in the pellet has a bright red color due to its heme content.

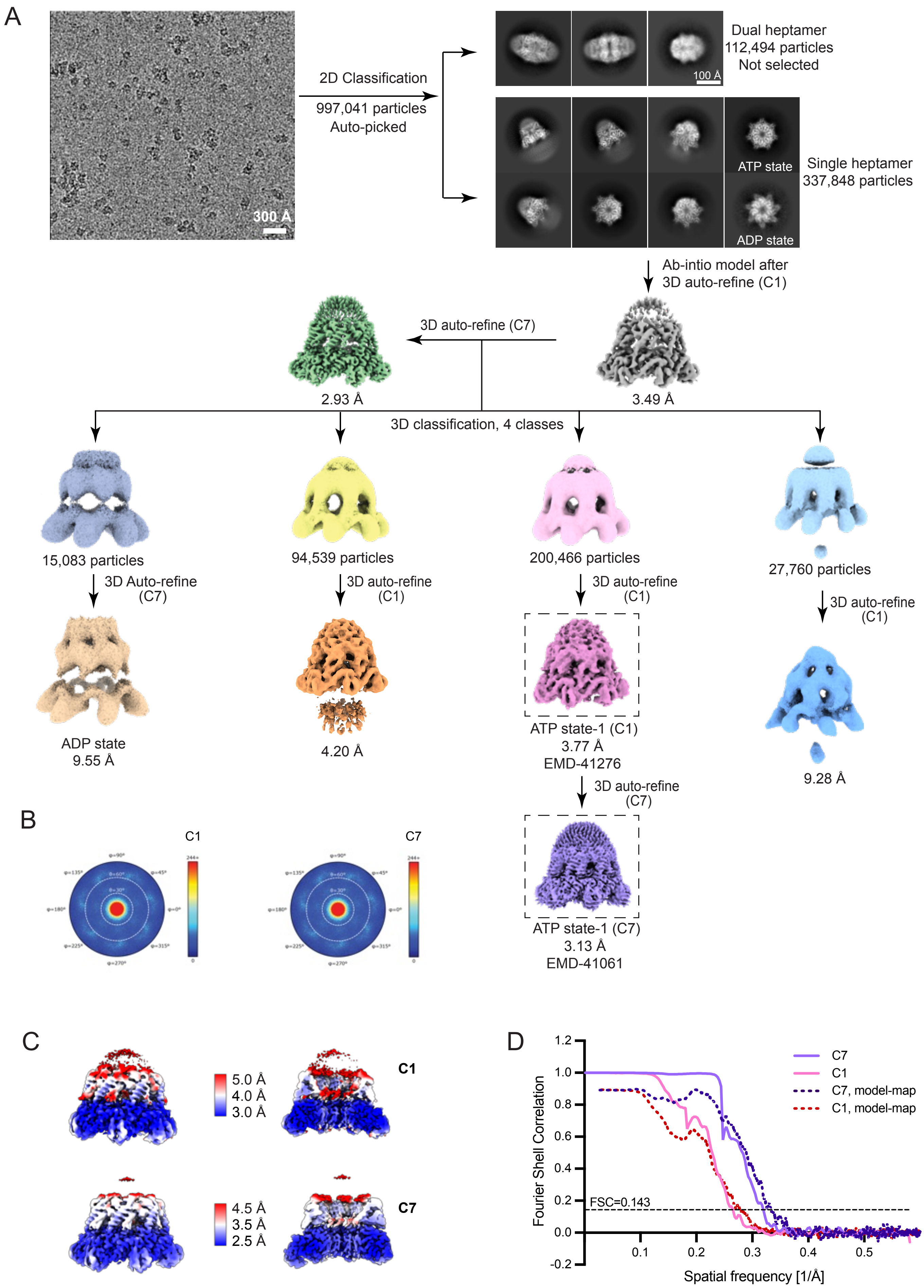

A
300 Å
2D Classification
997,041 particles
Auto-picked
100 Å
Dual heptamer
112,494 particles
Not selected
ATP state
ADP state
Single heptamer
337,848 particles
Ab-intio model after
3D auto-refine (C1)
3D auto-refine (C7)
2.93 Å
3.49 Å
3D classification, 4 classes
15,083 particles
94,539 particles
200,466 particles
27,760 particles
3D Auto-refine
(C7)
3D auto-refine
(C1)
3D auto-refine
(C1)
3D auto-refine
(C1)
ADP state
9.55 Å
4.20 Å
ATP state-1 (C1)
3.77 Å
EMD-41276
9.28 Å
3D auto-refine
(C7)
ATP state-1 (C7)
3.13 Å
EMD-41061
B
C1
C7
C
5.0 Å
4.0 Å
3.0 Å
C1
4.5 Å
3.5 Å
2.5 Å
C7
D
Fourier Shell Correlation
Spatial frequency [1/Å]
FSC=0.143
C7
C1
C7, model-map
C1, model-map
1.2
1.0
0.8
0.6
0.4
0.2
0.0
-0.2
0.1
0.2
0.3
0.4
0.5

Supplementary Figure 4. Data processing workflow and analysis for mBcs1L (ATP state-1) under ATP hydrolysis conditions in the absence of ISP-ED. (A) 2D classification of near 1 million auto-picked particles from 6,912 movies showed mBcs1L in solution formed single and dual heptamers. Only single heptamers were selected for further analysis. After 3D classification and refinement, both ADP and ATP conformations were obtained. (B) Angular distribution plots of all the particles that contributed to the final reconstruction in C1 symmetry (left) and C7 symmetry (right). (C) Local resolution maps for the ATP-bound mBcs1L (ATP state-1) structure processed in both C7 and C1 symmetry. Local resolution distribution of the entire structure is shown on the left and the same distribution in a cut-away is given on the right, displaying the local resolution distribution for the interior. (D) Gold-standard Fourier shell correlation (FSC) curves for the final density of the ATP-bound (ATP state-1) mBcs1L processed in both C7 and C1 symmetry are shown in solid lines. The reported resolutions for this structure were based on the FSC=0.143 criterion. FSC curves of the refined models versus their corresponding unfiltered and unsharpened maps are also shown in dashed lines.

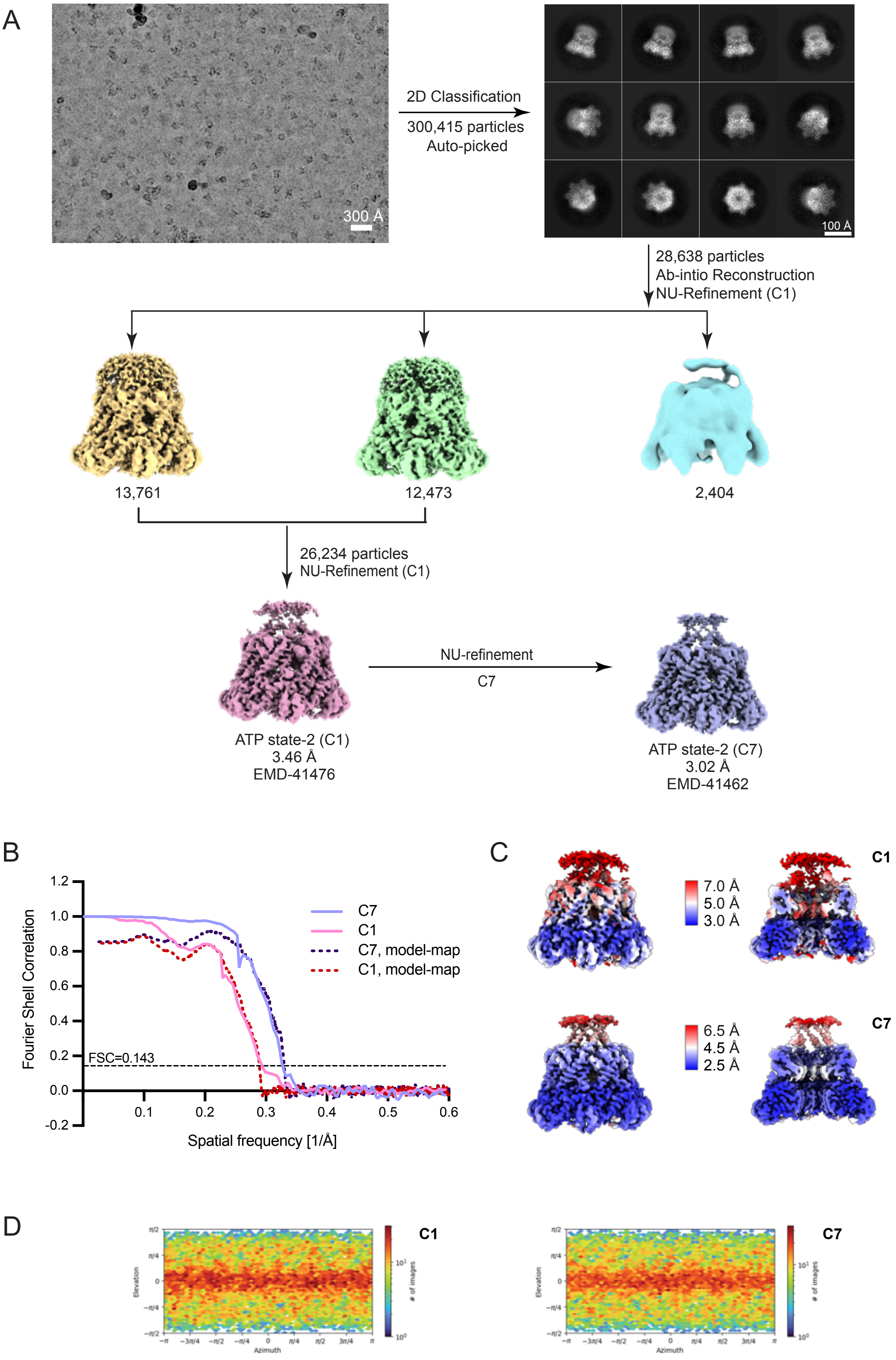
A
300 Å
2D Classification
300,415 particles
Auto-picked
100 Å
28,638 particles
Ab-intio Reconstruction
NU-Refinement (C1)
13,761
12,473
2,404
26,234 particles
NU-Refinement (C1)
NU-refinement
C7
ATP state-2 (C1)
3.46 Å
EMD-41476
ATP state-2 (C7)
3.02 Å
EMD-41462
B
Fourier Shell Correlation
Spatial frequency [1/Å]
FSC=0.143
C7
C1
C7, model-map
C1, model-map
C
7.0 Å
5.0 Å
3.0 Å
C1
6.5 Å
4.5 Å
2.5 Å
C7
D
C1
C7
Elevation
Azimuth
# of images

Supplementary Figure 5. Data processing workflow and analysis for the second smaller dataset of mBcs1L under ATP hydrolysis conditions (ATP state-2). (A) 2D class averages show mBcs1L particles formed mostly single heptamers instead of dual heptamers. After 3D reconstruction and classification, 26,234 particles were retained for final reconstruction using C1 or C7 symmetry. (B) Gold-standard Fourier shell correlation (FSC) curves of the final density of the mBcs1L in ATP state-2 processed in both C7 and C1 symmetry are shown in solid lines. FSC curves of the refined models versus their corresponding unfiltered and unsharpened maps are also shown in dashed lines. (C) Local resolution maps for the mBcs1L structure in ATP state-2 processed in both C7 and C1 symmetry. Local resolution distribution of the entire structure is shown on the left and the same distribution in a cut-away is given on the right, displaying the local resolution distribution for the interior. (D) Viewing direction distribution plots for the global non-uniform refinement map processed in C1 symmetry (left) and C7 symmetry (right).

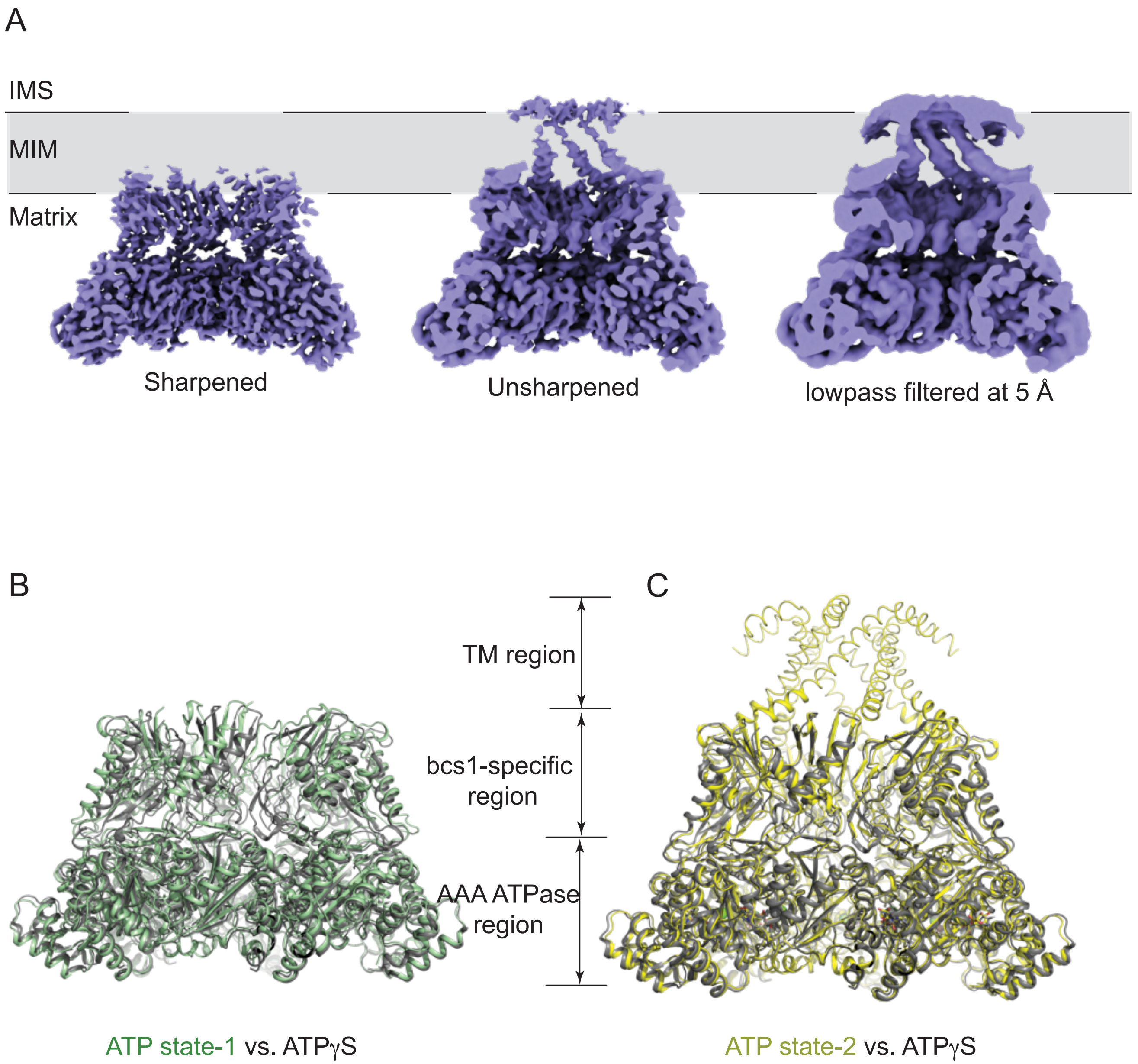
A
IMS
MIM
Matrix
Sharpened
Unsharpened
lowpass filtered at 5 Å
B
C
TM region
bcs1-specific
region
AAA ATPase
region
ATP state-1 vs. ATPγS
ATP state-2 vs. ATPγS

Supplementary Figure 6. EM density of ATP-bound mBcs1L in state-2 with various resolution filters. (A) Original unsharpened map (middle) was determined at 3.02 Å resolution using cisTEM. Auto-sharpened map (left) was used for modeling and permitted reliable sequence assignment. The lowpass-filtered map at 5 Å resolution (right) allowed better visualization of the TM helices. All maps were contoured at 5σ level. (B) Superposition of the model of mBcs1L in ATP state-1 (green) with that in ATPγS state (PDB:6UKS, gray). Three regions of the molecule are indicated. (C) Superposition of the model of mBcs1L in ATP state-2 (yellow) with that in ATPγS state (PDB:6UKS, gray).

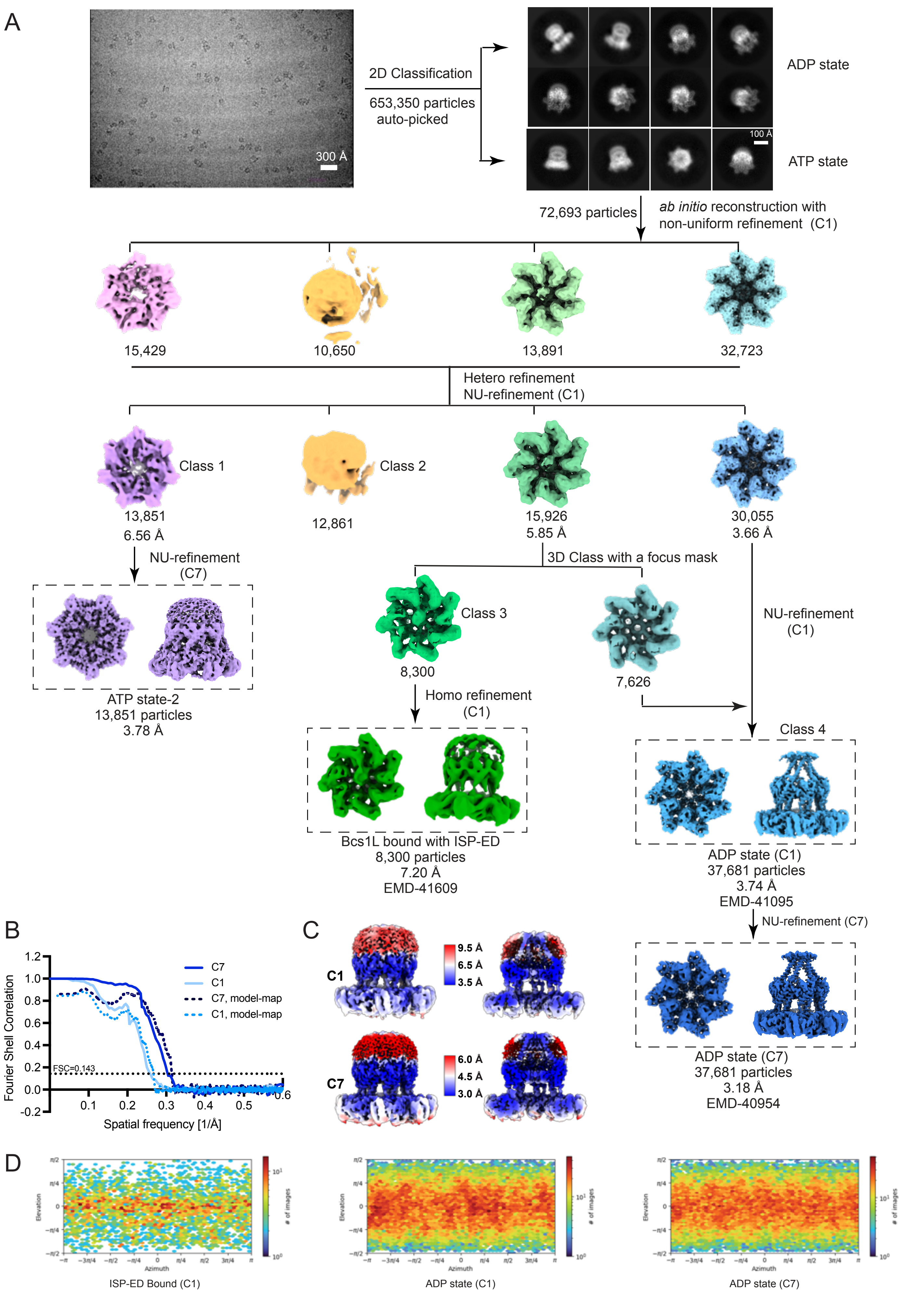

A
300 Å
2D Classification
653,350 particles auto-picked
ADP state
100 Å
ATP state
72,693 particles
ab initio reconstruction with non-uniform refinement (C1)
15,429
10,650
13,891
32,723
Hetero refinement
NU-refinement (C1)
Class 1
Class 2
13,851
6.56 Å
12,861
15,926
5.85 Å
30,055
3.66 Å
NU-refinement (C7)
3D Class with a focus mask
Class 3
8,300
7,626
NU-refinement (C1)
ATP state-2
13,851 particles
3.78 Å
Homo refinement (C1)
Class 4
Bcs1L bound with ISP-ED
8,300 particles
7.20 Å
EMD-41609
ADP state (C1)
37,681 particles
3.74 Å
EMD-41095
NU-refinement (C7)
B
Fourier Shell Correlation
Spatial frequency [1/Å]
C7
C1
C7, model-map
C1, model-map
FSC=0.143
C
C1
9.5 Å
6.5 Å
3.5 Å
C7
6.0 Å
4.5 Å
3.0 Å
ADP state (C7)
37,681 particles
3.18 Å
EMD-40954
D
Elevation
Azimuth
# of images
ISP-ED Bound (C1)
ADP state (C1)
ADP state (C7)

Supplementary Figure 7. Data processing workflow and analysis for mBcs1L under ATP hydrolysis conditions in the presence of ISP-ED. (A) 2D classification of auto-picked particles indicated a mixture of ADP state and ATP state particles. Further 3D reconstruction, classification and refinement revealed three distinct classes: ATP-2 state, ADP state with substrate-binding cavity empty and ADP state with ISP-ED bound. To separate ADP-state particles with and without ISP-ED bound, a focus mask on AAA-domain was used in the final 3D classification. (B) Gold-standard Fourier shell correlation (FSC) curves of the final density of the ADP-bound mBcs1L with empty substrate-binding cavity processed in both C7 and C1 symmetry are shown in solid lines. The reported resolutions for this structure were based on the FSC=0.143 criterion. FSC curves of the refined models versus their corresponding unfiltered and unsharpened maps are also shown in dashed lines. (C) Local resolution maps for the ADP-bound mBcs1L structure processed in both C7 and C1 symmetry. The local resolution distribution for the entire structure is shown on the left and the same distribution in a cut-away is given on the right, displaying the local resolution distribution for the interior. (D) Viewing direction distribution plots for the final ISP-ED bound map (left), ADP state map processed in C1 symmetry (middle) and C7 symmetry (right).

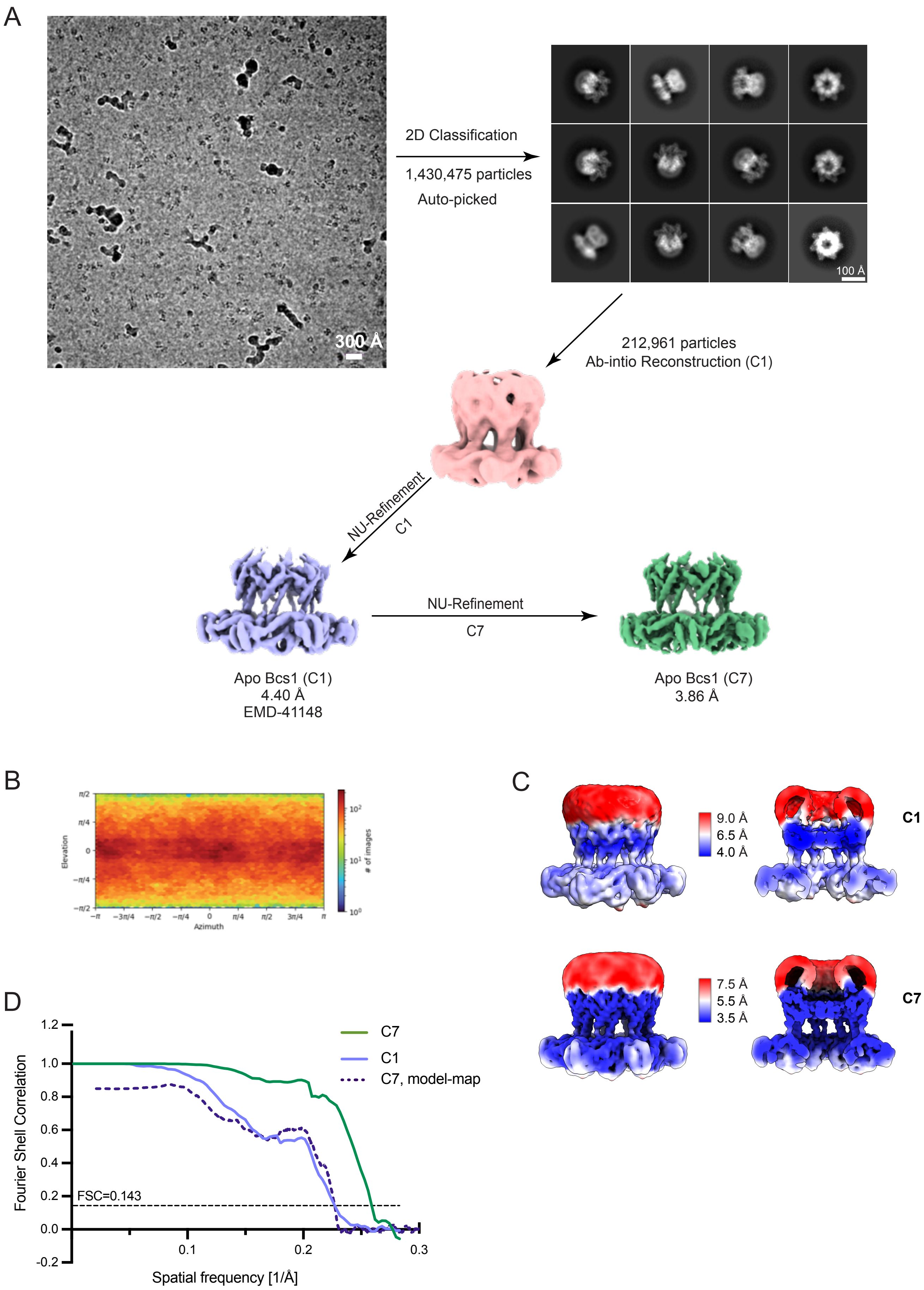
A
2D Classification
1,430,475 particles
Auto-picked
300 Å
100 Å
212,961 particles
Ab-intio Reconstruction (C1)
NU-Refinement
C1
NU-Refinement
C7
Apo Bcs1 (C1)
4.40 Å
EMD-41148
Apo Bcs1 (C7)
3.86 Å
B
Elevation
Azimuth
# of images
C
9.0 Å
6.5 Å
4.0 Å
C1
7.5 Å
5.5 Å
3.5 Å
C7
D
Fourier Shell Correlation
Spatial frequency [1/Å]
FSC=0.143
C7
C1
C7, model-map
1.2
1.0
0.8
0.6
0.4
0.2
0.0
-0.2
0.1
0.2
0.3

Supplementary Figure 8. Data re-processing workflow and analysis for mBcs1L under apo conditions. (A) 1,430,475 particles of mBcs1L were picked from 2,319 movies. After 2D classification, 212,961 particles were retained for reconstruction using C1 or C7 symmetry. (B) Viewing direction distribution plot for the final Apo Bcs1 map processed in C1 symmetry. (C) Local resolution maps for the apo mBcs1L structure processed in both C7 and C1 symmetry. Left panel shows the overall resolution distribution and the right panel shows the same distribution in a cut-away, displaying the local resolution distribution for the mBcs1L interior. (D) Gold-standard Fourier shell correlation (FSC) curves of the final density of the apo mBcs1L processed in both C7 and C1 symmetry are shown in solid lines. The reported resolutions for this structure were based on the FSC=0.143 criterion. FSC curve of the refined model versus the corresponding unfiltered and unsharpened map is also shown in dashed line.

A

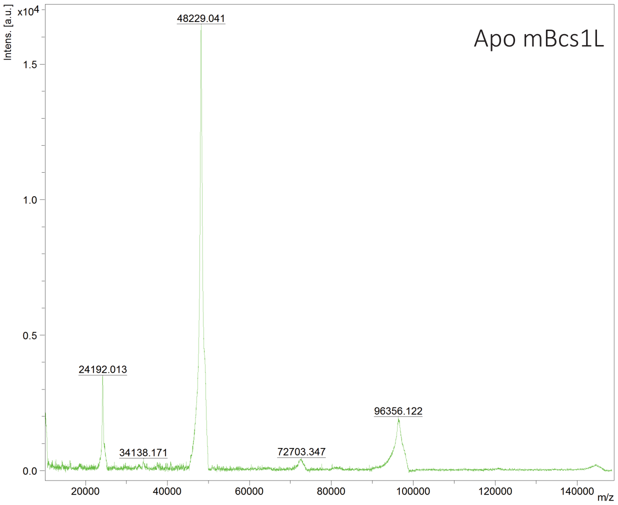

Apo mBcs1L
Intens. [a.u.]
x10^4
48229.041
24192.013
34138.171
72703.347
96356.122
0.0
0.5
1.0
1.5
20000
40000
60000
80000
100000
120000
140000
m/z

B

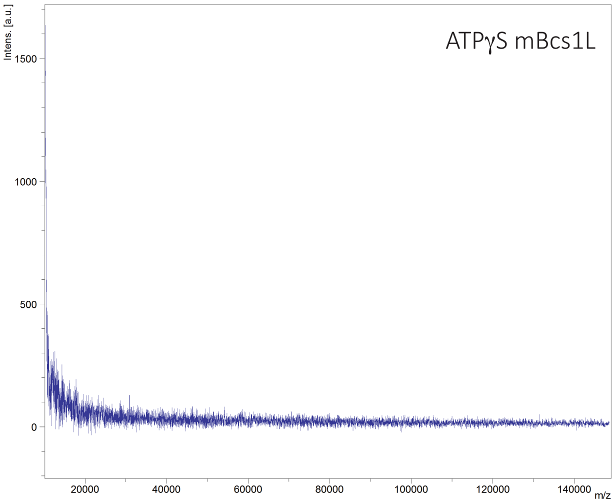

ATPγS mBcs1L
Intens. [a.u.]
0
500
1000
1500
20000
40000
60000
80000
100000
120000
140000
m/z

Supplementary Figure 9. Mass spectrometry of mBcs1L in the presence or absence of nucleotide. (A) Mass spectrometry profile for apo mBcs1L. (B) Mass spectrometry profile for mBcs1L in the presence of ATPγS.

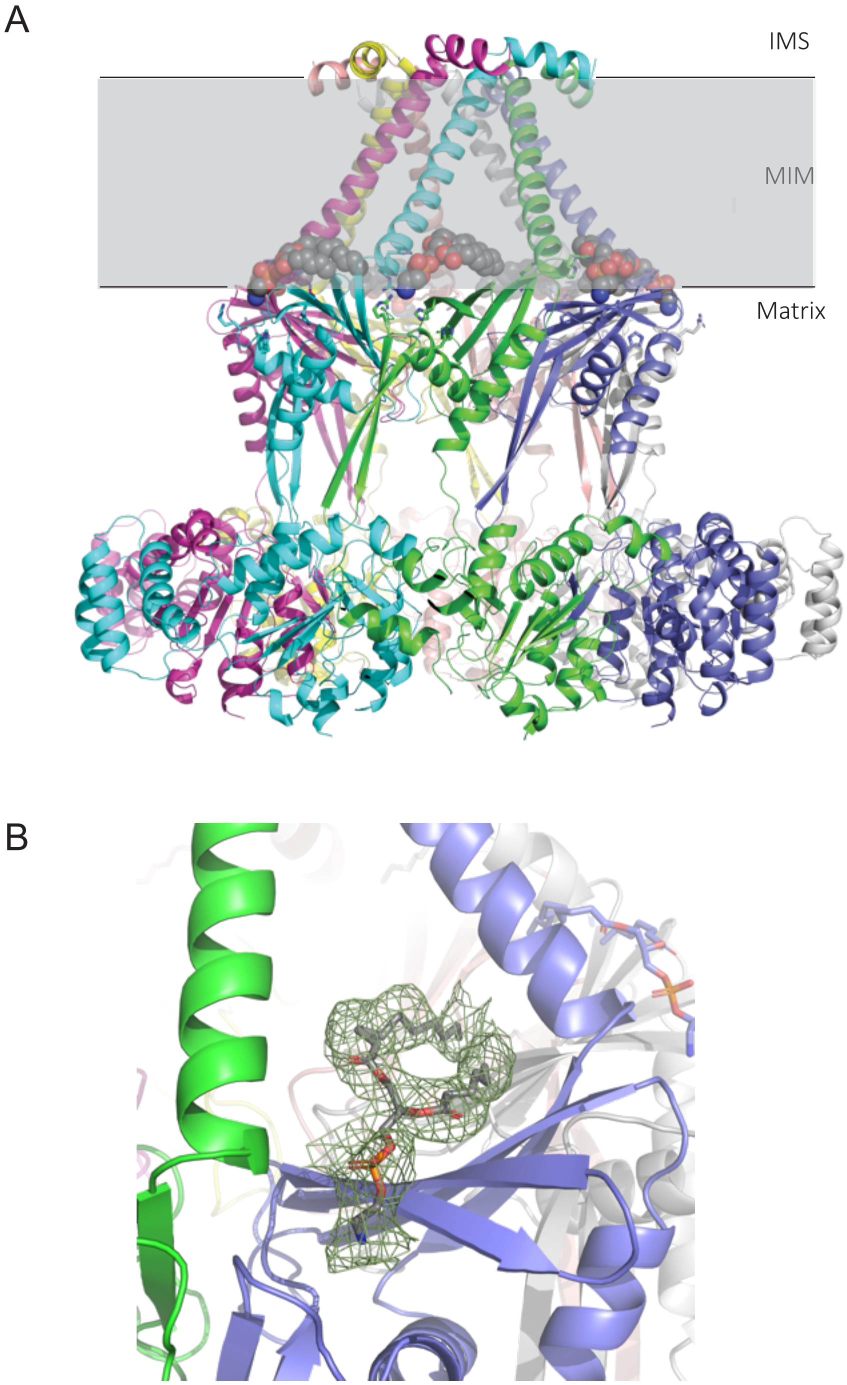
A
IMS
MIM
Matrix
B

Supplementary Figure 10. Association of lipid molecules seen with mBcs1L in the ADP conformation. (A) There are EM densities remaining in the TM region unassigned, especially in the C7 maps, after the completion of the model building for Bcs1 subunits. Some of these densities can be interpreted as lipids as shown here where PE lipid head groups were built into densities intercalating between TM helices on the matrix side of the mitochondrial inner membrane. (B) EM density unassigned in the C7 map after completion of heptameric Bcs1 model building. This density is rendered as cage wire in green and is contoured at 4σ. The density is fit with a lipid with PE head group.

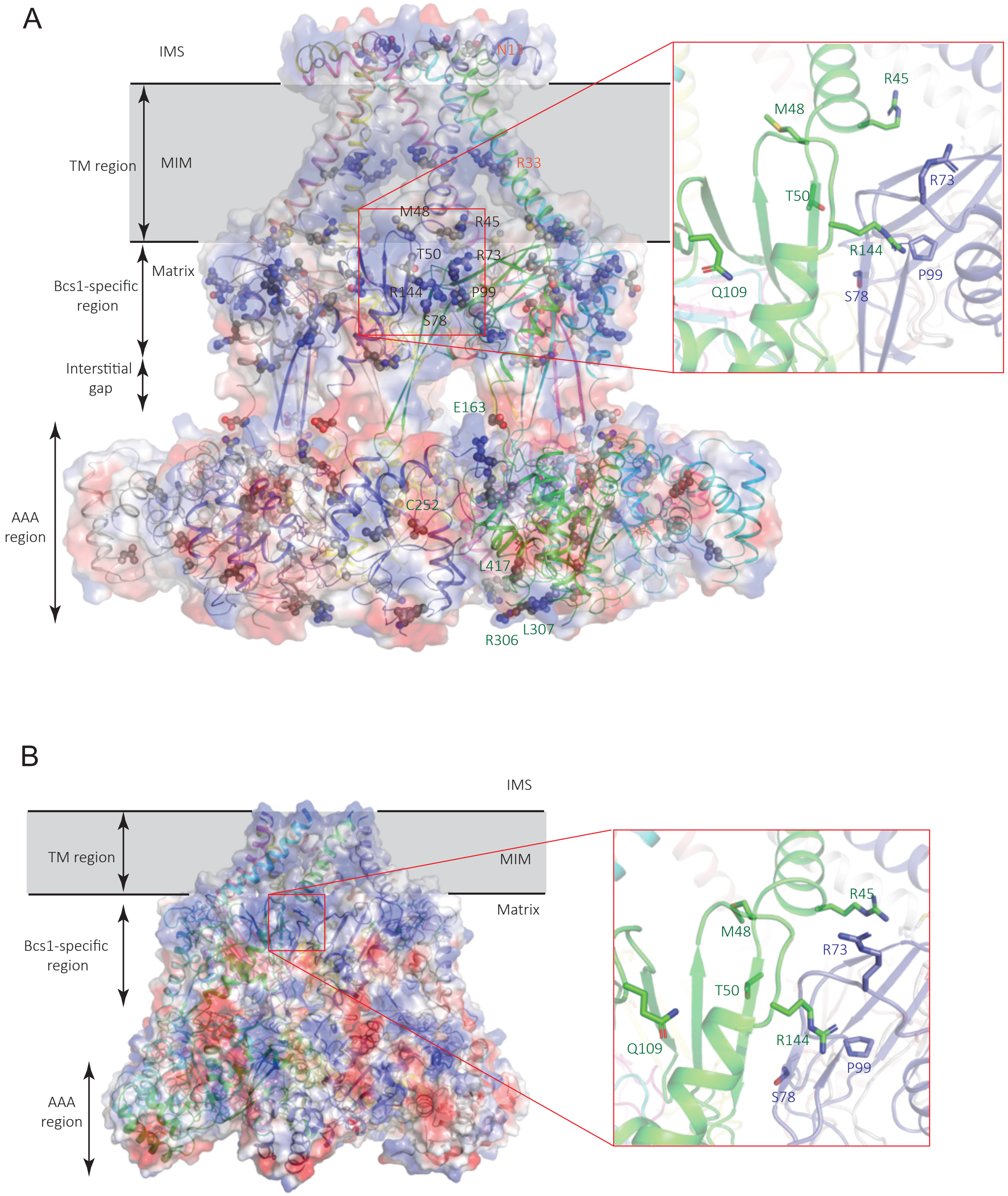
A
IMS
N13
TM region
MIM
R33
M48
R45
T50
R73
Matrix
Bcs1-specific region
R144
P99
S78
Interstitial gap
E163
AAA region
C252
L417
L307
R306
R45
M48
R73
T50
R144
Q109
P99
S78
B
IMS
TM region
MIM
Matrix
Bcs1-specific region
AAA region
R45
M48
R73
T50
Q109
R144
P99
S78

Supplementary Figure 11. Mapping of pathogenic mutations to Bcs1L structure and clustering of mutations associated with GRACILE syndrome. (A) The complete structure of heptameric mBcs1L in ADP-bound forms are rendered in cartoon form overlaid with an electrostatic potential surface, which has the positive potential in blue, negative potential in red, and neutral surface colored white. The mitochondrial inner membrane is demarcated with two horizontal black lines and a shaded rectangle. Definitions for various structural regions of the molecule are also given. 25 different pathogenic missense mutations, excluding those of deletions and terminations, are mapped to this structure as sphere models, some of which are labeled. The square inset on the right represents a small part of the Bcs1-specific region that borders the mitochondrial inner membrane (MIM) and at the interface between two subunits. The eight GS-linked mutations are rendered as stick models and labeled. (B) Structure of heptameric Bcs1L in the ATP-bound form. Only those mutations in the Bcs1-specific domain are shown and are magnified in the inset showing clustering of these mutations.
