# Supplementary material for: Conformations of Bcs1L undergoing ATP hydrolysis suggest a concerted translocation mechanism for folded iron-sulfur protein substrate
Source: Nat Commun. 2024 May 31;15:4655. doi: 10.1038/s41467-024-49029-y (PMC11143374; doi:10.1038/s41467-024-49029-y)
Supplement: Supplementary file 3 — Description of Additional Supplementary Files [file 41467_2024_49029_MOESM3_ESM.pdf]

File name: Supplementary Movie 1
Description: Viewed parallel to the membrane, the heptameric mBcs1L in the Apo state undergoes dramatic conformational change upon ATP binding showing the compression of the molecule. The molecule is re-oriented to be viewed down the 7-fold axis into the membrane showing the conformational change again from the Apo to the ATP bound state.
