# Supplementary material for: Conformations of Bcs1L undergoing ATP hydrolysis suggest a concerted translocation mechanism for folded iron-sulfur protein substrate
Source: Nat Commun. 2024 May 31;15:4655. doi: 10.1038/s41467-024-49029-y (PMC11143374; doi:10.1038/s41467-024-49029-y)
Supplement: Supplementary file 5 — Reporting Summary [file 41467_2024_49029_MOESM5_ESM.pdf]

nature portfolio

Corresponding author(s): Di Xia

Last updated by author(s): Apr 29, 2024

Reporting Summary

Nature Portfolio wishes to improve the reproducibility of the work that we publish. This form provides structure for consistency and transparency in reporting. For further information on Nature Portfolio policies, see our Editorial Policies and the Editorial Policy Checklist.

Statistics

For all statistical analyses, confirm that the following items are present in the figure legend, table legend, main text, or Methods section.

n/a	Confirmed	
☐	☒	The exact sample size (*n*) for each experimental group/condition, given as a discrete number and unit of measurement
☐	☒	A statement on whether measurements were taken from distinct samples or whether the same sample was measured repeatedly
☒	☐	The statistical test(s) used AND whether they are one- or two-sided<br>*Only common tests should be described solely by name; describe more complex techniques in the Methods section.*
☒	☐	A description of all covariates tested
☒	☐	A description of any assumptions or corrections, such as tests of normality and adjustment for multiple comparisons
☐	☒	A full description of the statistical parameters including central tendency (e.g. means) or other basic estimates (e.g. regression coefficient) AND variation (e.g. standard deviation) or associated estimates of uncertainty (e.g. confidence intervals)
☒	☐	For null hypothesis testing, the test statistic (e.g. *F*, *t*, *r*) with confidence intervals, effect sizes, degrees of freedom and *P* value noted<br>*Give P values as exact values whenever suitable.*
☒	☐	For Bayesian analysis, information on the choice of priors and Markov chain Monte Carlo settings
☒	☐	For hierarchical and complex designs, identification of the appropriate level for tests and full reporting of outcomes
☒	☐	Estimates of effect sizes (e.g. Cohen's *d*, Pearson's *r*), indicating how they were calculated

Our web collection on statistics for biologists contains articles on many of the points above.

Software and code

Policy information about availability of computer code

Data collection	SerialEM v3.8, SCIEX OS software v3
Data analysis	Graphpad Prism v9, cisTEM (beta v1.0), cryoSPARC (V4.2.1), Phenix (v1.20.1-4487), COOT (0.9.8), ChimeraX (v1.6.1), PyMOL (V2.4), ESPript v3.0, SCIEX OS software v3, Refmac7.0.076

For manuscripts utilizing custom algorithms or software that are central to the research but not yet described in published literature, software must be made available to editors and reviewers. We strongly encourage code deposition in a community repository (e.g. GitHub). See the Nature Portfolio guidelines for submitting code & software for further information.

Data

Policy information about availability of data

All manuscripts must include a data availability statement. This statement should provide the following information, where applicable:

- Accession codes, unique identifiers, or web links for publicly available datasets
- A description of any restrictions on data availability
- For clinical datasets or third party data, please ensure that the statement adheres to our policy

The Cryo-EM density maps and atomic models generated in this study have been deposited in the Electron Microscopy Data (EMD) Bank and in Protein Data Bank (PDB) under accession codes EMDB 41276 [https://www.ebi.ac.uk/emdb/EMD-41276] and PDB 8TI0 [https://doi.org/10.2210/pdb8TI0/pdb] (mBcs1L-ATP state-1 in C1); EMDB 41061 [https://www.ebi.ac.uk/emdb/EMD-41061] and PDB 8T5U [https://doi.org/10.2210/pdb8T5U/pdb] (mBcs1L-ATP state-1 in C7); EMDB 41476

[https://www.ebi.ac.uk/emdb/EMD-41476] and PDB 8TPL [https://doi.org/10.2210/pdb8TPL/pdb] (mBcs1L-ATP state-2 in C1); EMDB 41462 [https://www.ebi.ac.uk/emdb/EMD-41462] and PDB 8TP1 [https://doi.org/10.2210/pdb8TP1/pdb] (mBcs1L-ATP state-2 in C7); EMDB 41095 [https://www.ebi.ac.uk/emdb/EMD-41095] and PDB 8T7U [https://doi.org/10.2210/pdb8T7U/pdb] (mBcs1L-ADP state in C1); EMDB 40954 [https://www.ebi.ac.uk/emdb/EMD-40954] and PDB 8T14 [https://doi.org/10.2210/pdb8T14/pdb] (mBcs1L-ADP state in C7); EMDB 41609 [https://www.ebi.ac.uk/emdb/EMD-41609] (mBcs1L bound with ISP-ED), and EMDB 41148 [https://www.ebi.ac.uk/emdb/EMD-41148] and PDB 8TBY [https://doi.org/10.2210/pdb8TBY/pdb] (mBcs1L-Apo state in C1). Source data are provided with this paper.

Research involving human participants, their data, or biological material

Policy information about studies with human participants or human data. See also policy information about sex, gender (identity/presentation), and sexual orientation and race, ethnicity and racism.

Reporting on sex and gender	N/A
Reporting on race, ethnicity, or other socially relevant groupings	N/A
Population characteristics	N/A
Recruitment	N/A
Ethics oversight	N/A

Note that full information on the approval of the study protocol must also be provided in the manuscript.

Field-specific reporting

Please select the one below that is the best fit for your research. If you are not sure, read the appropriate sections before making your selection.

☒ Life sciences ☐ Behavioural & social sciences ☐ Ecological, evolutionary & environmental sciences

For a reference copy of the document with all sections, see nature.com/documents/nr-reporting-summary-flat.pdf

Life sciences study design

All studies must disclose on these points even when the disclosure is negative.

Sample size	EM particle sample size was determined by the automated particle picking and curation. The particles selected after rounds of 2D and 3D classification are sufficient to yield density maps with their resolutions determined by gold-standard FSC analysis .<br>Sample sizes for biochemical experiments were not predetermined. The sample sizes used are in line with conventions in the field and were sufficient to represent the reproducibility of the experiments.
Data exclusions	Only EM particles providing the highest quality 3D reconstruction were retained. Other particles were excluded. No data were excluded from biochemical experiments.
Replication	Cryo-EM maps were calculated from thousands of particles. Each Cryo-EM dataset was processed in both cryoSPARC and cisTEM software and reached consistent results.<br>Sample preparation experiments, including purification, SDS-PAGE, Western blot and ATPase activity measurement were successfully repeated at least three times. The mass spectrometry experiments were not replicated.
Randomization	For cryo-EM analysis, particles were randomly partitioned into two halves, each of which was used to obtain a 3D half-map independently in cisTEM and cryoSparc.
Blinding	Not applicable. No subjects or organisms that require blinding were analyzed.

Reporting for specific materials, systems and methods

We require information from authors about some types of materials, experimental systems and methods used in many studies. Here, indicate whether each material, system or method listed is relevant to your study. If you are not sure if a list item applies to your research, read the appropriate section before selecting a response.

Materials & experimental systems

n/a	Involved in the study
☐	☒ Antibodies
☐	☒ Eukaryotic cell lines
☒	☐ Palaeontology and archaeology
☒	☐ Animals and other organisms
☒	☐ Clinical data
☒	☐ Dual use research of concern
☒	☐ Plants

Methods

n/a	Involved in the study
☒	☐ ChIP-seq
☒	☐ Flow cytometry
☒	☐ MRI-based neuroimaging

Antibodies

Antibodies used

HisProb-HRP (Thermo Fisher Scientific, PI15165, 1:5,000 dilution).
Anti-Rieske bundle (Santa Cruz Biotechnology, sc-529220 with 1:1,000 dilution for the primary antibody and 1:10,000 dilution for he secondary antibody).

Validation

The primary Rieske FeS antibody is a mouse monoclonal IgG1 κ Rieske FeS antibody, is recommended for detection of Rieske FeS of mouse, rat and human origin by WB, IP, IF, IHC(P) and ELISA. It has been validated by the manufacturer using HeLa whole cell lysate, Neuro-2A whole cell lysate and NRK whole cell lysate. A recent publication (PMID: 37487647) also described the use of this primary Rieske FeS antibody.

HisProb-HRP (Thermo Fisher Scientific, PI15165) is a nickel (Ni2+)-activated derivative of horseradish peroxidase (HRP) that enables direct, IMAC-based detection of His-tagged proteins and other histidine-rich proteins in Western blots and microplates.

Eukaryotic cell lines

Policy information about cell lines and Sex and Gender in Research

Cell line source(s)

Pichia pastoris yeast strain, as available commercially and detailed in Materials and Methods

Authentication

The yeast strains were validated by the manufacturer.

Mycoplasma contamination

Not tested.

Commonly misidentified lines
(See ICLAC register)

No commonly misidentified cell lines were used.

Plants

Seed stocks

N/A

Novel plant genotypes

N/A

Authentication

N/A
